# Performance and outcome of ressucitative thoracotomies in a southern Brazil trauma center: a 7-year retrospective analysis

**DOI:** 10.1590/0100-6991e-20223146

**Published:** 2022-03-11

**Authors:** VITOR MAMORU HAIDA, EDUARDO MASSARO YAMASHITA, GIÓRGIA SOUZA FRANCO, WELLIDHA BIANCA ROCHA AMADO, ISABELLA KOHATSU ARAKAKI, CAROLINE LOUISE BALCEWICZ DAL-BOSCO, JAQUELINE ALVES ZWIERZIKOWSKI, IWAN AUGUSTO COLLAÇO, GUILHERME PASQUINI CAVASSIN

**Affiliations:** 1 - Universidade Positivo, Departamento de Medicina - Curitiba - PR - Brasil; 2 - Hospital do Trabalhador, Departamento de Cirurgia Geral - Curitiba - PR - Brasil; 3 - Universidade Federal do Paraná, Departamento de Medicina - Curitiba - PR - Brasil

**Keywords:** Thoracotomy, Wounds and Injuries, Thoracic Surgical Procedures, Trauma Severity Indices, Toracotomia, Ferimentos e Lesões, Procedimentos Cirúrgicos Torácicos, Índices de Gravidade do Trauma

## Abstract

**Objective:**

the study aims to analyze the performance and outcome of resuscitation thoracotomy (TR) performed in patients victims of penetrating and blunt trauma in a trauma center in southern Brazil during a 7 years period.

**Methods:**

retrospective study based on the analysis of medical records of patients undergoing TR, from 2014 to 2020, in the emergency service of the Hospital do Trabalhador, Curitiba - Paraná, Brazil.

**Results:**

a total of 46 TR were performed during the study period, of which 89.1% were male. The mean age of patients undergoing TR was 34.1±12.94 years (range 16 and 69 years). Penetrating trauma corresponded to the majority of indications with 80.4%, of these 86.5% victims of gunshot wounds and 13.5% victims of knife wounds. On the other hand, only 19.6% undergoing TR were victims of blunt trauma. Regarding the outcome variables, 84.78% of the patients had declared deaths during the procedure, considered non-responders. 15.22% of patients survived after the procedure. 4.35% of patients undergoing TR were discharged from the hospital, 50% of which were victims of blunt trauma.

**Conclusion:**

the data obtained in our study are in accordance with the world literature, reinforcing the need for a continuous effort to perform TR, respecting its indications and limitations in patients victims of severe penetrating or blunt trauma.

## RESUMO

**Objetivo:**

analisar o desempenho e o desfecho das toracotomias de reanimação (TR) realizadas nos pacientes vítimas de trauma penetrante e contuso em um hospital de referência em trauma no Sul do Brasil durante um período de sete anos.

**Métodos:**

estudo retrospectivo baseado na análise de prontuários de pacientes submetidos a TR, no período de 2014 a 2020, no serviço de emergência do Hospital do Trabalhador, Curitiba - Paraná, Brasil.

**Resultados:**

um total de 46 TR foram realizadas durante o período de estudo, dos quais 89.1% eram do sexo masculino. A média de idade dos pacientes submetidos a TR foi de 34.1±12.94 anos (variação de 16 e 69 anos). O trauma penetrante correspondeu pela maioria das indicações de TR com 80.4%, destas 86.5% vítimas de ferimentos por arma de fogo e 13.5% vítimas de ferimento por arma branca. Por outro lado, apenas 19.6% submetidos a TR foram vítimas de trauma contuso. No que se refere as variáveis de desfecho, 84.78% dos pacientes tiveram óbitos declarados durante o procedimento, considerados não respondedores. 15.22% dos pacientes apresentaram sobrevida após o procedimento. 4.35% dos pacientes submetidos à TR tiveram alta hospitalar, sendo 50% pacientes vítimas de trauma contuso.

**Conclusão:**

os dados obtidos em nosso estudo estão em conformidade com a literatura mundial, reforçando a necessidade de um esforço contínuo para realização da TR respeitando suas indicações e limitações em pacientes vítimas de trauma grave penetrante ou contuso.

## INTRODUCTION

Resuscitative thoracotomy (RT), a procedure performed to manage patients in extreme conditions, has remained among the most polarizing and controversial surgical acts performed by physicians since its first formal description in the 1960s^1^ ^,^ ^2^. Currently, there is unquestionable indication of RT in patients sustaining chest injuries with absence of pulse, but who maintain signs of life, such as extremity movement, cardiac electrical activity, pupillary response, among others. However, the use of RT after blunt trauma is less understood and remains controversial due to low survival rates^3^ ^,^ ^4^. When RT is used, therapeutic goals include hemorrhage control, clamping of the pulmonary hilum in case of air embolism or massive bronchopleural fistula, cardiac tamponade relief, and clamping of the descending aorta to control hemorrhage^5^.

When making the decision to perform RT, injury mechanism, location of the injury, and signs of life must be analyzed, as they directly contribute to a greater probability of a positive outcome6. In this sense, the highest survival rate is associated with penetrating injuries and the presence of vital signs on admission^7^. On the other hand, a systematic review involving 27 studies showed that only 1.5% of patients who survived the procedure had good neurological recovery (without functional or cognitive impairment)^3^. In another systematic review, Rhee et al.^7^ described overall survival after RT between 1.8% and 27.5%. Survival rates reported after RT vary widely, which may be due to different characteristics between pre-hospital services, population distribution, mechanism of injury, and sample size^8^. 

Thus, the present study aims to retrospectively analyze the performance and outcome of RT in victims of penetrating and blunt trauma in a trauma referral hospital in Southern Brazil, in the last 7 years. 

## METHODS

### Study design and period

We conducted a retrospective, observational, and descriptive study that analyzed the medical records of patients undergoing RT, from 2014 to 2020, in the emergency service of the Hospital do Trabalhador, Curitiba - Paraná state, Brazil.

### Definitions

We defined RT as an immediate thoracotomy performed on an emergency basis in the emergency room or in the operating room in patients with absence of pulse after blunt or penetrating trauma, with or without signs of life^1^. The following parameters were considered as signs of life: pupillary response, spontaneous breathing, presence of carotid pulse, measurable or palpable blood pressure, extremity movement, and cardiac electrical activity^9^. Thus, we included patients who met the proposed definition, and excluded those with insufficient data in their medical records.

The Injury Severity Score (ISS) is an anatomical trauma score that considers the affected anatomical region and the degree of severity in each region, ranging from 1 to 5. Furthermore, the most severe injury score of the three most injured segments is squared, with trauma being classified as mild (ISS <9), moderate (ISS 9-14), and severe (ISS >14), ranging from 1 to 75^10^. In cases where a segment is classified as an untreatable lesion (6), the score automatically assumes a value of 75^10^. 

The Revised Trauma Score (RTS) uses the Glasgow Coma Scale (GCS), respiratory rate, and systolic blood pressure, ranging from 0 to 12. A lower score predicts lower survival^11^. 

The Glasgow Coma Scale (GCS) is used to identify a patient’s level of consciousness, assessing the capacity for motor response, verbal response, and eye opening, ranging from 3 to 15 points^12^.

The Trauma Injury Severity Score (TRISS) is a method used in the retrospective analysis of the survival probability of trauma patients, which uses a mathematical equation including RTS, ISS, and patient age^13^. In addition, it allows to determine the quality of service provided at the institution and to compare it with that of other trauma centers^13^. 

### Variables analyzed

We assessed sex, trauma mechanism, clinical data at admission, outcome, postoperative complications, mortality, and hospital discharge. For the classification of trauma severity, we used ISS, TRISS, RTS, and GCS.

### Statistical analysis

We analyzed quantitative variables with the Mann-Whitney test, and qualitative ones, with the Fisher’s exact test. We performed all statistical tests with the GRAPHPAD PRISM^®^ statistical package, considering a significance level of 5% (p<0.05).

### Research Ethics

The Ethics in Research Committee approved the study with the opinion 4.369.508, on October 29^th^, 2020. Ethics Assessment Presentation Certificate (CAAE) 39591220.0.0000.5225.

## RESULTS

**Figure 1:**
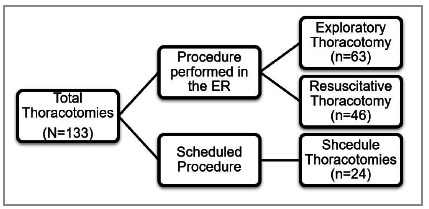
Thoracotomies performed during the 2014-2020 period.

Of 133 thoracotomies analyzed, 46 included RT (Figure 1). Among the RT, 89.1% of patients were male. The mean age was 34.1 ± 12.9 years (range 16-69). There was a predominance of the young population, with 69.5% of patients aged ≤40 years. Penetrating trauma was responsible for most RT indications (80.4%), of which 86.5% were due to gunshot wounds (GSW), and 13.5%, stab wounds (SW). On the other hand, 19.6% of individuals undergoing RT were victims of blunt trauma (Table 1).

**Table 1:** Epidemiology and clinical characteristics of deaths and hospital discharges after resuscitative thoracotomy.

Variable	Total (n=46)	Death (n=44)	Discharge (n=2)	p-value
Sex (n;%)				
Male	41 (89.1)	40 (90.9)	1 (50)	0.208
Female	5 (10.9)	4 (9.1)	1 (50)	
Age (Average; Standard deviation; range)	34.1 ± 12.9 (16-69)	34.11 ± 13.2 (16-69)	34.00 ±.07 (29-39)	0.846
Trauma Mechanism (n;%)				
Blunt	9 (19.5)	8 (18.2)	1 (50)	0.364
Penetrating	37 (80.5)	35 (79.5)	1 (50)	
Clinical Parameters at admission (n;%)				
GCS <12	39 (84.8)	39 (88.6)	0	0.020*
GCS 13-15	7 (15.2)	5 (11.4)	2 (100)	
SBP ≥75	15 (32.6)	13 (29.5)	2 (100)	0.101
SBP <75	31 (70.4)	31 (70.4)	0	
RTS (Average; Standard Deviation; Range)	2.63 ± 2.97 (0.0 -7.84)	2.40 ± 2.82 (0.0-7.84)	7.70 ± 0.21 (7.55-7.84)	0.015*
TRISS (Average; Standard deviation; Range)	31.10 ± 40.64 (0.01-99.39)	28.08 ± 38.91 (0.1-99.4)	97.45 ± 1.62 (96.3-98.6)	0.031*
ISS (Average; Standard deviation; Range)	29.96 ± 13.58 (9-66)	30.27 ± 13.79 (9-66)	23.00 ± 4.24 (20-26)	0.556
GCS (Average; Standard Deviation; Range)	5.83±4.32 (3-15)	5.45±4.02 (3-15)	14±1.41 (13-15)	0.020*

ISS: injury severity score. TRISS: trauma injury severity score. RTS: revised trauma score. GCS: glasgow coma scale. SBP: systolic blood pressure. *p<0.05.

**Figure 2:**
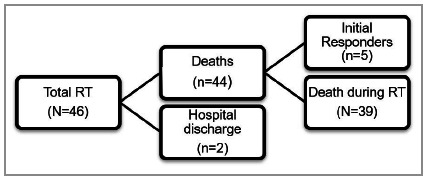
Clinical outcomes of patients undergoing resuscitative thoracotomy.

Regarding outcome, 84.8% of patients died during the procedure, and were considered non-responders. On the other hand, RT responders (those who responded to initial resuscitation measures and came out alive after the operation) had a survival rate after the procedure of 15.2%. However, 71.4% of responding patients died (Table 2). Therefore, we obtained a hospital discharge rate of 4.4% (Figure 2).

**Table 2:** Clinical characteristics of responders to resuscitative thoracotomy. .

Variable	Responders (n=7)
Sex (n;%)	
Masculine	6 (85.7)
Feminine	1 (14.3)
Age (Average; Standard deviation; Range)	31.1±12.1
Trauma Mechanism (n;%)	
Blunt	1 (14.3)
Penetrating	6 (85.7)
Clinical Parameters on admission (n;%)	
GCS <12	3 (42.8)
GCS 13-15	4 (57.2)
SBP ≥75	4 (57.2)
SBP <75	3 (42.8)
RTS (Average; Standard Deviation; Range)	4.33±3.51 (0-7 ± 841)
TRISS (Average; Standard deviation; Range)	49.71±46.12 (0.4-98.6)
ISS (Average; Standard deviation; Range)	25.85±11.71 (9-45)
Complications after RT (n;%)	
hemodynamic instability	3 (60)
(<24 hours)	
Sepsis	1 (20)
(After 24 hours)	
Brain death	1 (20)

ISS: injury severity score. TRISS: trauma injury severity score. RTS: revised trauma score. GCS: glasgow coma scale. SBP: systolic blood pressure.

## DISCUSSION

In the last 40 years, indications for RT have been improved due to bettered information on results and outcomes after this procedure^14^. Thus, although there is great heterogeneity of indications and applications evidenced by the wide variability in survival rates reported in the medical literature, currently RT has been associated with excellent results in reducing mortality in specific circumstances^=^. 

According to the 2015 recommendations of the American College of Surgeons, RT should be used in patients with signs of life and absence of pulse after a penetrating chest injury. However, RT is conditionally recommended for pulseless patients who show absent signs of life after chest injury, for those with vital signs present or absent after penetrating extrathoracic injury, or those with signs of life present after blunt injury. Also conditionally, RT is not recommended for patients with absence of pulse and vital signs after blunt injury^1^. Furthermore, as limiting factors for performing RT, some authors point out the restricted resources in the emergency room, the costs inherent to the procedure, and the risk of exposure to blood-borne pathogens, such as HIV^16^ ^,^ ^17^. However, the study by Baker, Thomas, and Trunkey^18^ showed that the total benefits of RT were 2.4 times greater than the total cost of the procedure, taking into account economic aspects such as shorter hospital stay and low cost when compared with other procedures. In parallel, the study by Branney, Moore, and Feldhaus^19^ also demonstrated a favorable cost-benefit ratio for RT. In addition, a study published in 2018 concluded that the risk of exposure of the staff involved in RT is low and that it should not influence the decision to perform the procedure^20^. 

In accordance with the literature^8^ ^,^ ^16^, most patients undergoing RT in our study were male (89.1%) and belonged to the young age group, with a mean age of 34.1 years. Similar to our results, the sample from the Brazilian study by Guimarães et al. showed that 89.5% of patients undergoing RT were male and the mean age was 29.2 years^15^. As for the trauma mechanism, our study showed the predominance of penetrating trauma. In this sense, although there are unicentric studies, such as the one by Thorsen et al.^8^ carried out in Norway, which showed a predominance of blunt trauma, ours agrees with the results found in the systematic review by Nevins et al., with 37 articles jointly evaluating more than 3,000 patients undergoing RT^21^. 

According to DiGiacomo and Angus, RT is a highly morbid intervention that should be used in extremely severe cases^16^. The overall survival rate indicated in the study by the American College of Surgeons in 2001 was 7.8%, despite a systematic review carried out in 2020 showing overall survival rates between 0 and 50%^1^ ^,^ ^21^. We observed a death rate during the procedure of 84.8% and a survival rate after RT of 15.2%. The meta-analysis by Rhee et al. and the systematic review by Nevins et al. reported survival rates after RT in blunt trauma of 1.4% and 5.2%, respectively^7^ ^,^ ^21^. In our study, we had a hospital discharge rate of 2.1% after RT in blunt trauma. For some authors, the mortality rate associated with RT is probably due to the patient’s critical condition, as well as to the indication for the procedure^22^. 

In line with the literature, our results on ISS, RTS, GCS, and TRISS were more favorable in patients who survived. Onat et al.^2^ had a mean of 30.71 points in patients who underwent RT and died. This result is similar to what we found (30.27 points). Regarding patients who responded to RT, Ito et al.^24^ reported a mean ISS of 25, a value similar to that found in our analysis (25.85). 

Higher RTS values indicate patients with better clinical conditions^11^. In our study, patients who were discharged from the hospital had a mean RTS of 7.70. On the other hand, patients who died had a mean of 2.63. These results agree with those of Thorsen et al.^8^, who found a mean RTS value for the group that died of 2, and for the group that survived, of 8. 

As for the Glasgow Coma Scale, according to Lustenberger et al.^25^ a GCS greater than 8 is an independent predictor of survival after RT. In agreement, we observed a mean GCS in patients who died of 5.45, and for those who were discharged from the hospita^l,14^. Similarly, Thorsen et al.^8^ observed that patients who died and those who survived after RT had mean GCS values of 2 and 12, respectively. However, the systematic review by Narvestad et al.^26^ indicated that most of the studies analyzed did not use GCS or another objective scale for the neurological assessment of patients undergoing RT. In that review, only a subjective qualitative assessment to characterize the patient’s neurological status was reported. Thus, in the right patient population, RT cannot only save lives, but also potentially lead to good recovery and function after otherwise fatal injuries^27^. 

The unicentric analysis, relatively small sample, retrospective evaluation, and absence of previously defined sample size were limitations of our study. Thus, larger-sample, multicenter, and prospective studies are needed for further elucidation of RT.

## CONCLUSION

The analysis of RT performed at a trauma referral center in southern Brazil points to results similar to those reported in the world literature. Data from our study support the need for continuous effort to perform RT, respecting the indications and limitations in victims of severe penetrating or blunt trauma. 
